# Comparison of Two Strategies for Prophylactic Donor Lymphocyte Infusion in Patients With Refractory/Relapsed Acute Leukemia

**DOI:** 10.3389/fonc.2021.554503

**Published:** 2021-03-03

**Authors:** Qiongqiong Su, Zhiping Fan, Fen Huang, Na Xu, Danian Nie, Dongjun Lin, Ziwen Guo, Pengcheng Shi, Zhixiang Wang, Ling Jiang, Jing Sun, Zujun Jiang, Qifa Liu, Li Xuan

**Affiliations:** ^1^ Department of Hematology, Nanfang Hospital, Southern Medical University, Guangzhou, China; ^2^ Department of Hematology, Sun Yat-Sen Memorial Hospital, Sun Yat-Sen University, Guangzhou, China; ^3^ Department of Hematology, The Third Affiliated Hospital, Sun Yat-Sen University, Guangzhou, China; ^4^ Department of Hematology, Zhongshan People’s Hospital, Zhongshan, China; ^5^ Department of Hematology, Guangzhou General Hospital of Guangzhou Military Command, Guangzhou, China

**Keywords:** prophylactic donor lymphocyte infusion, refractory/relapsed acute leukemia, relapse, allogeneic hematopoietic stem cell transplantation, minimal residual disease

## Abstract

Prophylactic donor lymphocyte infusion (pDLI) could reduce relapse in patients with refractory/relapsed acute leukemia (RRAL) undergoing allogeneic hematopoietic stem cell transplantation (allo-HSCT), but optimal timing of pDLI remains uncertain. We compared the outcomes of two strategies for pDLI based on time from transplant and minimal residual disease (MRD) status in patients with RRAL. For patients without grade II–IV acute graft-versus-host disease (aGVHD) on day +60, pDLI was given on day +60 regardless of MRD in cohort 1, and was given on day +90 unless MRD was positive on day +60 in cohort 2. A total of 161 patients with RRAL were enrolled, including 83 in cohort 1 and 78 in cohort 2. The extensive chronic GVHD (cGVHD) incidence in cohort 2 was lower than that in cohort 1 (10.3% vs. 27.9%, *P* = 0.006) and GVHD-free/relapse-free survival (GRFS) in cohort 2 was superior to that in cohort 1 (55.1% vs. 41.0%, *P* = 0.042). The 2-year relapse rate, overall and leukemia-free survival were comparable between the two cohorts (29.0% vs. 28.2%, *P* = 0.986; 63.9% vs. 64.1%, *P* = 0.863; 57.8% vs. 61.5%, *P* = 0.666). Delaying pDLI to day +90 based on MRD for patients with RRAL undergoing allo-HSCT could lower extensive cGVHD incidence and improve GRFS without increasing incidence of leukemia relapse compared with pDLI on day +60.

## Introduction

Allogeneic hematopoietic stem cell transplantation (allo-HSCT) is accepted as the optimal choice for patients with refractory/relapsed acute leukemia (RRAL) (1, 2). However, relapse remains a barrier for the survival of these refractory patients post-transplant, with incidences of relapse of over 50% and leukemia-free survival (LFS) of about 25% (3, 4). Some studies have demonstrated that prophylactic donor lymphocyte infusion (pDLI) is effective for preventing relapse in patients with RRAL post-transplant (5–8), but its complication of graft-versus-host disease (GVHD) has limited its application (9, 10). The morbidity and mortality of GVHD post-pDLI are related with the time interval between pDLI administration and transplantation as well as the doses and donor source of pDLI (11–13), but optimal timing of pDLI remains unknown. Our previous prospective multicenter study showed that pDLI on day +60 post-transplant regardless of minimal residual disease (MRD) could reduce relapse for patients with RRAL undergoing allo-HSCT, but the 2-year cumulative incidences of extensive chronic GVHD (cGVHD) and mortality of GVHD reached up to 21.1% and 14.1% (7).

In order to reduce the morbidity and mortality of GVHD post-pDLI, we modified our pDLI strategy by delaying the time to day +90 unless MRD was positive on day +60. We aimed at evaluating whether this new strategy for pDLI could reduce the morbidity and mortality of GVHD post-pDLI but not affect relapse and survival in patients with RRAL undergoing allo-HSCT compared with our history strategy.

## Materials and Methods

### Study Population

This study was based on two prospective, independent and non-parallel cohorts. Cohort 1 came from a non-registered prospective multicenter study (7), and cohort 2 from a registered prospective multicenter clinical trial (NCT02673008). Patients undergoing allo-HSCT between January 2012 and December 2017 were enrolled in this study if they met the following criteria: (1) patients with RRAL without complete remission (CR) pre-transplant, including patients with acute myeloid leukemia (AML), acute lymphoblastic leukemia (ALL), and acute biphenotypic leukemia (ABL); (2) achieving CR at 30 days post-transplant; (3) with available donor lymphocytes; (4) no evidence of relapse, uncontrolled infection, or serious organ failure at the time of the planned pDLI. RRAL was defined as primary induction failure after two or more cycles of chemotherapy or relapse refractory to salvage chemotherapy (14, 15). Enrolled patients who were not treated with pDLI due to factors such as GVHD were also included in this study. This study was approved by respective ethical review boards before study initiation, and written informed consent was obtained from all patients in accordance with the Declaration of Helsinki.

### Transplantation

The sequential intensified conditioning regimen was administered in all patients: fludarabine 30 mg/m^2^/day and cytarabine 2 g/m^2^/day (on days −10 to −6), 4.5 Gy total body irradiation/day (on days −5 and −4), and cyclophosphamide 60 mg/kg/day and etoposide 15 mg/kg/day (on days −3 and −2). All patients undergoing HLA-matched sibling donor (MSD) or HLA-matched unrelated donor (MUD) transplant received peripheral blood stem cell (PBSC) grafts whereas patients undergoing HLA-haploidentical donor (HID) transplant received a combination of bone marrow (BM) and PBSC grafts.

### Graft-Versus-Host Disease Prophylaxis and Immunosuppressant Withdrawal

Ciclosporin A (CsA) alone or CsA + methotrexate (MTX) were administered in patients undergoing MSD transplant, and CsA + MTX + antithymocyte globulin and/or mycophenolate were used in patients receiving MUD or HID transplant for GVHD prophylaxis (16, 17). Immunosuppressant was withdrawn gradually in patients without acute GVHD (aGVHD) by day +30, and was stopped at 90 days after MSD transplant or 120 days after HID or MUD transplant if patients had no GVHD. For patients receiving pDLI before day +90 after allo-HSCT, immunosuppressant was continued for another 2 weeks after pDLI, then tapered and stopped within 4 weeks if no DLI-associated GVHD occurred. If patients had GVHD, immunosuppressant was reduced by 50% when GVHD was controlled and then stopped 2 weeks later.

### pDLI

**Figure 1:**
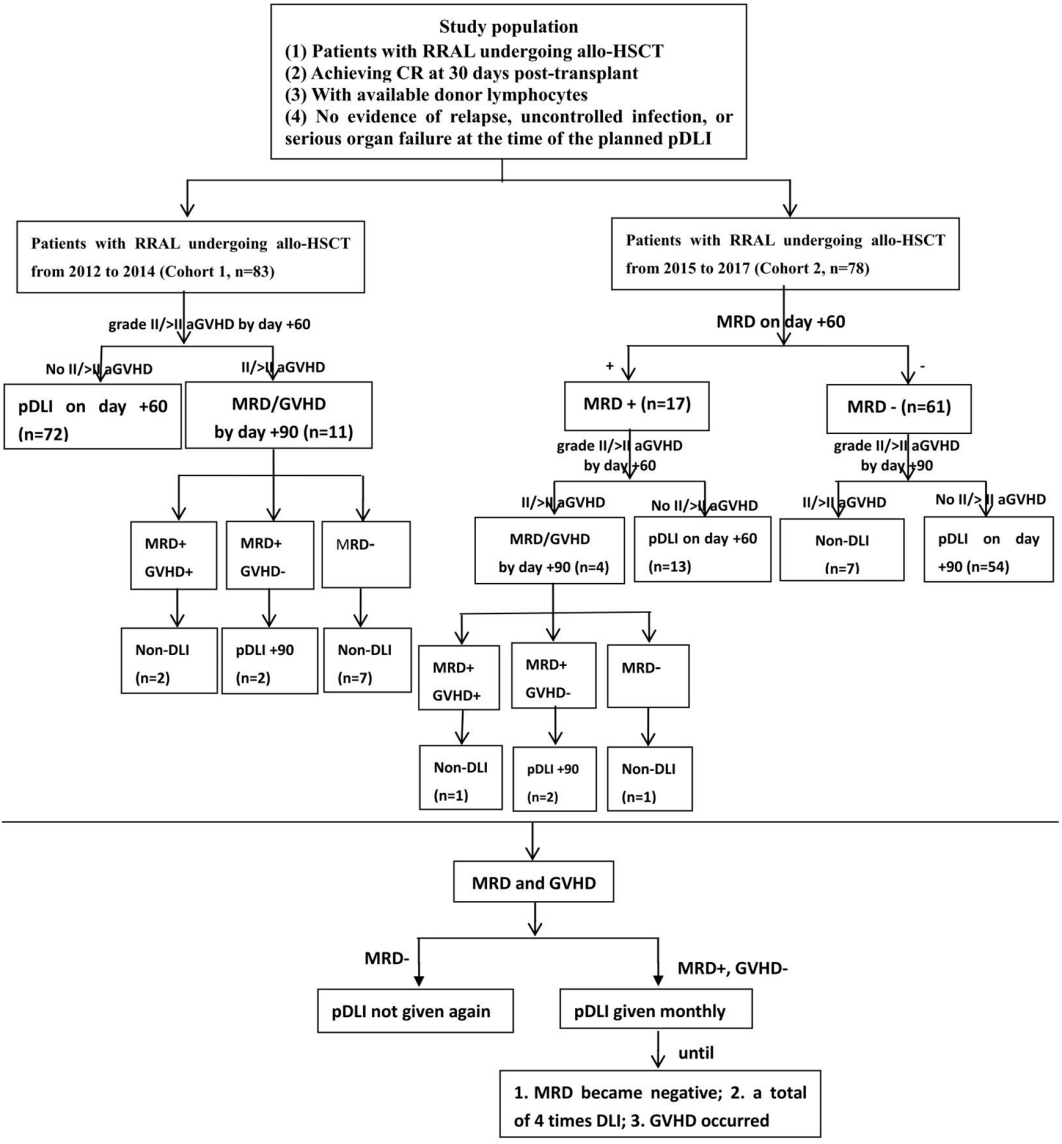
Protocol of two pDLI strategies for patients with RRAL undergoing allo-HSCT. RRAL, refractory/relapsed acute leukemia; allo-HSCT, allogeneic hematopoietic stem cell transplantation; CR, complete remission; pDLI, prophylactic donor lymphocyte infusion; aGVHD, acute graft-versus-host disease; MRD, minimal residual disease.

pDLI used granulocyte colony-stimulating factor (G-CSF)-mobilized PBSCs (G-PBSCs), which were derived from previously cryopreserved or newly collected G-PBSCs. The CD3^+^ T cell count for each pDLI was 3.0 × 10^7^/kg of the recipient weight. pDLI strategies of the two cohorts are conducted as shown in **Figure 1**. In cohort 1, pDLI was given once on day +60 regardless of MRD for all patients without grade II–IV aGVHD, and then administered based on MRD and GVHD status. If patients were MRD negative, pDLI was not given again; if patients were MRD positive and without grade II–IV aGVHD, pDLI was given monthly until GVHD occurred or MRD became negative or for a total of four times. For patients with grade II or above aGVHD by day +60 post-transplant, the application of pDLI was based on MRD and GVHD status by day +90. If patients remained MRD positive and had no GVHD on day +90, pDLI was given once on day +90 and then administered based on MRD and GVHD status. In cohort 2, for patients who were MRD negative on day +60 and did not experience grade II–IV aGVHD by day +90, pDLI was given once on day +90 post-transplant and then administered based on MRD and GVHD status. For patients with positive MRD and without grade II–IV aGVHD on day +60, pDLI was given once on day +60 and then administered based on MRD and GVHD status. For patients with positive MRD and grade II–IV aGVHD on day +60, the application of pDLI was based on the MRD and GVHD status by day +90. If patients remained MRD positive and had no GVHD on day +90, pDLI was given once on day +90 and then administered based on MRD and GVHD status.

### Surveillance and Intervention for Relapse

BM samples were analyzed pre-transplant and then once a month in the first 6 months post-transplant, once every 2 months from 6^th^ to 12^th^, once every 3 months from 13^th^ to 24^th,^ and once every 4 months from the 25^th^ to 36^th^ post-transplant for the monitoring of morphology and MRD. If MRD was positive, it was monitored once a week until MRD became negative. Aberrant leukemia-associated immune phenotypes detected by 8-color flow cytometry (FCM) and leukemia-related genes detected by polymerase chain reaction (PCR) were used for MRD test. FCM positive was defined as >0.01% of cells with leukemia-associated aberrant immune phenotypes. Leukemia-related fusion genes including AML1/ETO, CBFβ/MYH11 and BCR/ABL were tested and the threshold for PCR positivity was ≥ 0.001%. Subjects were scored as MRD positive if they had two consecutive positive results using FCM or PCR or were both FCM and PCR positive in a single BM sample (7, 18).

### Evaluation Points and Definitions

The primary endpoint was cGVHD. Secondary endpoints included aGVHD, relapse, overall survival (OS), LFS, GVHD-free/relapse-free survival (GRFS), and non-relapse mortality (NRM). aGVHD and cGVHD were graded as described previously (19, 20). CR was defined as <5% blasts in the BM and no persistence of extramedullary disease. Relapse was defined as reappearance of leukemic blasts in peripheral blood or ≥5% blasts in BM or reappearance or new appearance of extramedullary leukemia. OS was defined as the time from transplantation until death from any cause. LFS was defined as the time from transplantation until relapse or death from any cause. NRM was defined as death from any cause not subsequent to relapse. GRFS was a composite endpoint of allo-HSCT, comprising grade III–IV aGVHD, cGVHD requiring systemic immunosuppressive treatment, NRM and relapse, and represented real recovery after transplantation.

### Statistics

Our study data were analyzed on June 30, 2019. Statistical analyses were performed using SPSS 20.0 (SPSS Inc., Chicago, IL, USA) and R version 3.3.0 (R Development Core Team, Vienna, Austria). The chi-square and Mann-Whitney U tests were used for categorical and continuous variables, respectively. OS, LFS, and GFRS were estimated using Kaplan-Meier method and compared using log-rank test. Cumulative incidences of relapse, NRM and GVHD were calculated by accounting for competing risks. Competing risks for GVHD included death without GVHD and relapse. Relapse and NRM were competing risks for each other. The Cox proportional hazards model was used for the analysis of risk factors for time-to-event variables. Strategy, number, and donor source of pDLI were included in the multivariable analyses for GVHD in pDLI recipients. The following variables were included in the univariable analyses for relapse and survival: gender, patient age, disease category, genetic status, BM blasts on day 0, transplant modality, strategy and number of pDLI, aGVHD, and cGVHD. Only variables with *P* < 0.10 were included in the multivariable analyses for relapse and survival. All statistical tests were two-tailed with a significance level of 0.05.

## Results

### Patient Characteristics

A total of 161 patients with RRAL undergoing allo-HSCT from January 2012 to December 2017 were eligible for the study, including 69 patients with AML, 76 with ALL, and 16 with ABL. Eighty-three patients undergoing allo-HSCT from January 2012 to December 2014 and adopting previous pDLI strategy were enrolled in cohort 1, and 78 patients who underwent allo-HSCT from January 2015 to December 2017 and adopted modified pDLI strategy were enrolled in cohort 2. There were no significant differences between the two cohorts in sex, age, disease category, genetics, BM blasts at transplantation, transplant modality, and condition of tapering immunosuppressants (all *P* > 0.05) (**Table 1**).

**Table 1:** Patients’ clinical and transplant characteristics.

Patient characteristics	Cohort 1 (n = 83)	Cohort 2 (n = 78)	*P* value
Sex, Female/Male	25 (30.1%) /58 (69.9%)	34 (43.6%) /44 (56.4%)	0.076
Age, median (range), years	30 (12–57)	26 (14–51)	0.328
Disease category			0.215
AML	36 (43.4%)	33 (42.3%)	
ALL	42 (50.6%)	34 (43.6%)	
ABL	5 (6.0%)	11 (14.1%)	
Genetic			0.941
Favorable	4 (4.8%)	4 (5.1%)	
Intermediate	32 (38.6%)	28 (35.9%)	
Unfavorable	47 (56.6%)	46 (59.0%)	
Median BM blasts before conditioning (range)	32.0% (9.0%–91.0%)	35.0% (12.0%–93.0%)	0.725
Donor source			0.431
MSD	48 (57.8%)	39 (50.0%)	
MUD	14 (16.9%)	12 (15.4%)	
HID	21 (25.3%)	27 (34.6%)	
Stem cell source			0.197
PBSCs	62 (74.7%)	51 (65.4%)	
PBSCs + BM	21 (25.3%)	27 (34.6%)	
Median CD34+ cells per graft, 10^6^/kg (range)	9.01 (4.79–17.37)	8.64 (5.86–12.00)	0.815
Tapering immunosuppressants			
Withdrawing on day +30	62 (74.7%)	59 (75.6%)	0.890
Discontinuing on day +90 Discontinuing on day +120	14 (16.9%) 27 (32.5%)	16 (20.5%) 27 (34.6%)	0.553 0.779

AML, acute myeloid leukemia; ALL, acute lymphoblastic leukemia; ABL, acute biphenotypic leukemia; BM, bone marrow; MSD, HLA-matched sibling donor; MUD, HLA-matched unrelated donor; HID, HLA-haploidentical donor; PBSCs, peripheral blood stem cells.

### pDLI

Of the 161 patients included, 9 patients in cohorts 1 and 2 did not receive pDLI, respectively. In cohort 1, 74 patients (72 on day +60; 2 on day +90) underwent a total of 112 courses of pDLI, including 47 patients with 1 course, 19 with 2 courses, 5 with 3 courses and 3 with 4 courses, while 69 patients (13 on day +60; 56 on day +90) in cohort 2 received 102 courses of pDLI, including 46 patients with 1 course, 15 with 2 courses, 6 with 3 courses and 2 with 4 courses (*P* = 0.764). The median number of pDLI was 1 (range: 1–4) per patient, with no difference between the two cohorts (*P* = 0.170). The median CD3^+^ T cells of per pDLI was 3.0 (1.8–5.2) × 10^7^/kg and 3.0 (2.0–4.5) × 10^7^/kg in cohorts 1 and 2 (*P* = 0.317). In addition, the positive rates of MRD on day +60 and +90 post-transplant in cohort 1 were 19/83 (22.9%) and 10/83 (12.0%), compared with 17/78 (21.8%) and 11/78 (14.1%) in cohort 2 (*P* = 0.867, *P* = 0.699). The leukemia relapse rate from day +60 to +90 had no significant difference between the two cohorts (3.6% vs. 3.8%, *P* = 1.000).

### Graft-Versus-Host Disease

**Figure 2:**
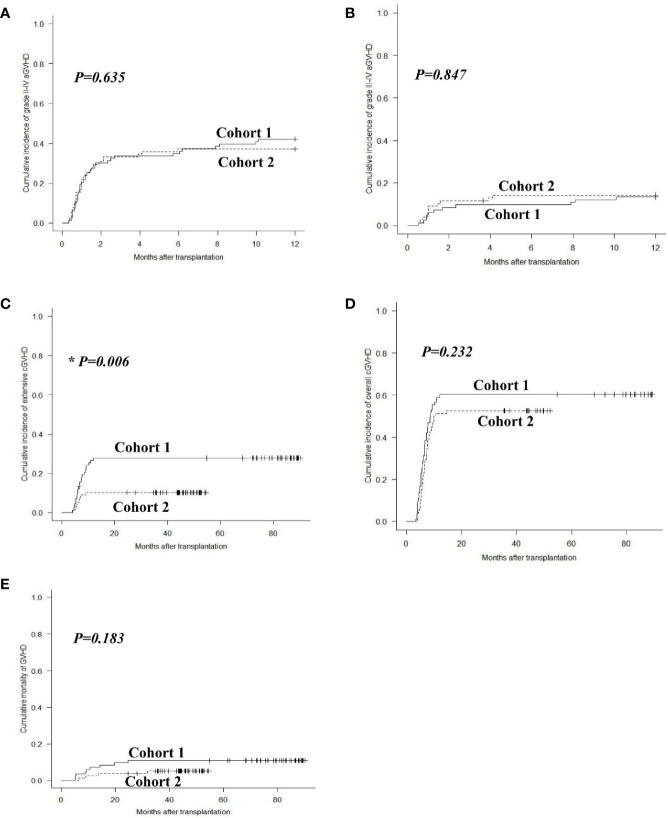
GVHD after allo-HSCT. Cumulative incidences of grade II–IV aGVHD **(A)**, grade III–IV aGVHD **(B)**, extensive cGVHD **(C)**, overall cGVHD **(D)** and mortality of GVHD **(E)** in cohorts 1 and 2.

The 1-year overall cumulative incidence of grade II–IV aGVHD was 42.2% (95% confidence interval (CI): 31.4%–52.6%) and 37.2% (26.5%–47.8%; *P* = 0.635), and grade III–IV aGVHD was 13.3% (95% CI: 7.0%–21.5%) and 14.1% (7.5%–22.9%; *P* = 0.847) in cohorts 1 and 2, respectively (**Figures 2A, B**). The 2-year extensive cGVHD incidence in cohort 2 [10.3% (95% CI: 4.8%–18.2%)] was lower than that in cohort 1 [27.9% (18.7%–37.9%)] (*P* = 0.006, **Figure 2C**). The 2-year overall cGVHD incidence was 60.2% (95% CI: 48.7%–69.9%) and 52.6% (40.8%–63.0%; *P* = 0.232), and GVHD mortality was 10.8% (95% CI: 5.3%–18.6%) and 5.2% (1.7%–11.8%; *P* = 0.183) in cohorts 1 and 2, respectively (**Figures 2D, E**).

The incidences of grade II–IV and III–IV aGVHD after pDLI showed no significant differences between the two cohorts (*P* = 0.428, *P* = 0.887). The extensive cGVHD incidence after pDLI in cohort 2 was lower than that in cohort 1 (9.0% vs. 28.6%, *P* = 0.004). The overall cGVHD incidence and GVHD mortality after pDLI were similar between the two cohorts (*P* = 0.177, *P* = 0.146). In multivariable analysis, increasing numbers of pDLI predicted higher incidences of grade II–IV and III–IV aGVHD (*P* = 0.028, hazard risk (*HR*) = 2.046; *P* = 0.020, *HR* = 3.690), and a trend toward a higher incidence of extensive cGVHD (*P* = 0.054). Additionally, the modified pDLI strategy was associated with a lower risk of extensive cGVHD compared with previous pDLI strategy (*P* = 0.011, *HR* = 0.306). Donor source of pDLI was not associated with the incidence of aGVHD or cGVHD (all *P* > 0.05) (**Table 2**).

**Table 2:** Multivariable analyses for risk factors of GVHD in pDLI recipients.

Parameters	Grade II–IV aGVHD		Grade III–IV aGVHD		Overall cGVHD		Extensive cGVHD	
	Hazard risk (95% CI)	*P* Value	Hazard risk (95% CI)	*P* Value	Hazard risk (95% CI)	*P* Value	Hazard risk (95% CI)	*P* Value
Strategy of pDLI modified vs. previous	0.798 (0.417–1.527)	0.496	1.095 (0.378–3.168)	0.868	0.835 (0.539–1.295)	0.420	0.306 (0.123–0.758)	*0.011
Number of pDLI >1 vs. 1	2.046 (1.079–3.879)	*0.028	3.690 (1.233–11.040)	*0.020	0.894 (0.561–1.425)	0.638	2.597 (0.983–6.866)	0.054
Donor source of pDLI MSD MUD HID	0.894 (0.611–1.307)	0.562	0.712 (0.390–1.300)	0.269	0.978 (0.754–1.269)	0.868	0.862 (0.556–1.337)	0.507

pDLI, prophylactic donor lymphocyte infusion; aGVHD, acute graft-versus-host disease; cGVHD, chronic graft-versus-host disease; MSD, HLA-matched sibling donor; MUD, HLA-matched unrelated donor; HID, HLA-haploidentical related donor; CI, confidence interval; *P < 0.05.

### Relapse

**Figure 3:**
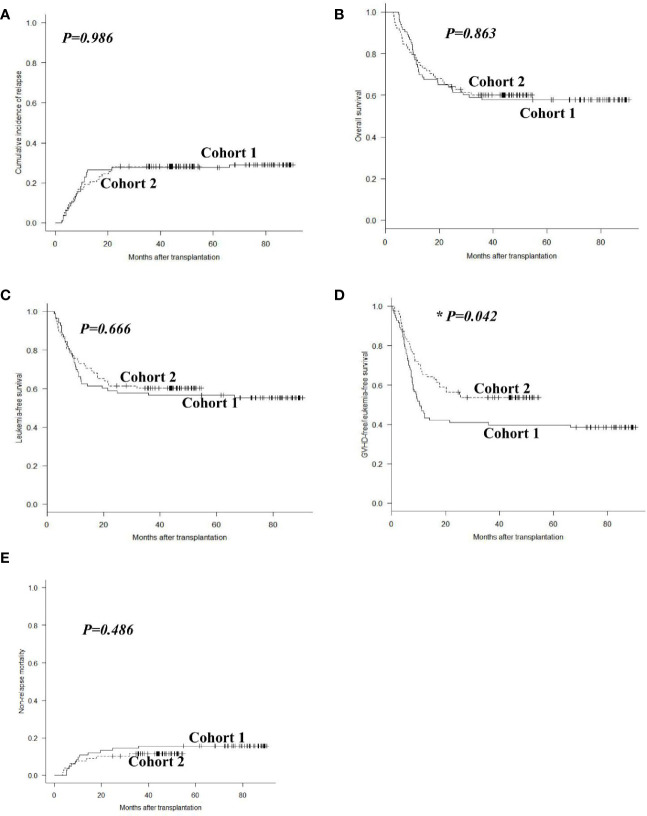
Outcomes after allo-HSCT. Cumulative incidences of relapse **(A)**, overall survival **(B)**, leukemia-free survival **(C)**, GVHD-free/relapse-free survival **(D)**, and non-relapse mortality **(E)** in cohorts 1 and 2.

In cohort 1, 24 patients experienced relapse at a median time of 243 (range: 71 to 1988) days post-transplant, including 17 hematological, 3 extramedullary, and 4 both hematological and extramedullary relapse. In cohort 2, 22 patients relapsed at a median time of 232 (range: 77 to 654) days post-transplant, with 16 hematological, 4 extramedullary and 2 both hematological and extramedullary relapse. The 2-year cumulative incidence of leukemia relapse was 29.0% (95% CI: 19.6%–39.0%) and 28.2% (18.7%–38.5%) in cohorts 1 and 2 (*P* = 0.986, **Figure 3A**). In multivariable analysis, HID transplant and cGVHD were protective factors for relapse (*P* = 0.038, *HR* = 0.476; *P* = 0.041, *HR* = 0.526), and the percentage of BM blasts ≥3% on day 0 was the only risk factor for relapse (*P* = 0.001, *HR* = 4.340) (**Table 3**).

**Table 3:** Univariable and multivariable analyses for risk factors of relapse and survival.

Parameters	Relapse		Overall survival		Leukemia-free survival		GVHD-free/relapse-free survival (GRFS)	
	Univariable *P* value	Multivariable *P* value;HR (95% CI)	Univariable *P* value	Multivariable *P* value;HR (95% CI)	Univariable *P* value	Multivariable *P* value; HR (95% CI)	Univariable *P* value	Multivariable *P* value; HR (95% CI)
Female vs. male	0.160	–	0.866	–	0.627	–	0.283	–
Patient age ≥29 vs. <29 years (median)	0.207	–	0.133	–	0.172	–	0.111	–
Disease category AML vs. ALL vs. ABL	0.644	–	0.759	–	0.657	–	0.736	–
Genetic status Other vs. unfavorable	0.201	–	0.451	–	0.318	–	0.253	–
BM blasts on day 0 ≥3% vs. <3% (median)	*0.001	*0.001; 4.340 (2.359–7.987)	*0.001	*0.001; 2.861 (1.744–4.693)	*0.001	*0.001; 3.016 (1.843–4.936)	*0.001	*0.001; 3.656 (2.328–5.743)
Transplant modality HID vs. MSD/MUD	*0.040	*0.038; 0.476 (0.237–0.959)	0.125	–	0.061	0.076; 0.610 (0.354–1.053)	0.222	–
Strategy of pDLI modified vs. previous	0.456	–	0.863	–	0.666	–	*0.044	*0.010; 0.459 (0.292–0.722)
Number of pDLI 0 vs. 1 vs. >1	0.552	–	0.514	–	0.375	–	0.776	–
aGVHD II–IV vs. 0–I	0.359	–	* 0.012	0.061; 1.604 (0.978–2.630)	*0.023	0.096; 1.517 (0.928–2.480)	*0.009	*0.020; 1.679 (1.086–2.596)
cGVHD vs. No cGVHD	*0.030	*0.041; 0.526 (0.294–0.939)	*0.002	*0.002; 0.454 (0.279–0.739)	*0.005	*0.010; 0.524 (0.321–0.855)	0.677	–

AML, acute myeloid leukemia; ALL, acute lymphoblastic leukemia; ABL, acute biphenotypic leukemia; BM, bone marrow; HID, HLA-haploidentical related donor; MSD, HLA-matched sibling donor; MUD, HLA-matched unrelated donor; pDLI, prophylactic donor lymphocyte infusion; aGVHD, acute graft-versus-host disease; cGVHD, chronic graft-versus-host disease; HR, hazard risk; CI, confidence interval; *P < 0.05.

### Survival

In cohort 1, 48 patients survived and 35 died with a median follow-up of 2,164 (range, 148 to 2,712) days post-transplant. Causes of death included leukemia relapse (n = 20), GVHD (n = 10), infections (n = 4), and others (n = 1). In cohort 2, 47 patients survived and 31 died with a median follow up of 1,108 (range, 91 to 1637) days post-transplant. Causes of death included leukemia relapse (n = 20), infections (n = 6), GVHD (n = 4), and others (n = 1). The 2-year OS and LFS were 63.9% (95% CI: 52.5%–73.1%) and 57.8% (46.5%–67.6%) in cohort 1, compared with 64.1% (95% CI: 52.4%–73.6%) and 61.5% (49.8%–71.3%) in cohort 2 (*P* = 0.863, *P* = 0.666, **Figures 3B, C**). However, the 2-year GRFS in cohort 2 was superior to that in cohort 1 (55.1% vs. 41.0%, *P* = 0.042, **Figure 3D**). The 2-year NRM was 13.2% (95% CI: 7.0%–21.5%) and 10.3% (4.8%–18.2%) in cohorts 1 and 2 (*P* = 0.486, **Figure 3E**). Multivariable analysis revealed that cGVHD was the only protective factor for OS and LFS (*P* = 0.002, *HR* = 0.454; *P* = 0.010, *HR* = 0.524), and modified pDLI strategy was the only protective factor for GRFS (*P* = 0.010, *HR* = 0.459). The percentage of BM blasts ≥3% on day 0 was the only risk factor for OS and DFS (*P* = 0.001, *HR* = 2.861; *P* = 0.001, *HR* = 3.016); the percentage of BM blasts ≥3% on day 0 and grade II–IV aGVHD were risk factors for GRFS (*P* = 0.001, *HR* = 3.656; *P* = 0.020, *HR* = 1.679) (**Table 3**).

## Discussion

Several studies including ours have shown that pDLI could prevent relapse in patients with RRAL undergoing allo-HSCT (5–8, 21). However, the high morbidity and mortality of GVHD post-pDLI have limited its application (9, 10). The morbidity and mortality of GVHD post-pDLI are related with the time of pDLI post-transplant (5, 11, 13). In this study, we compared the outcomes of two strategies for pDLI based on time from transplant and MRD status post-transplant in patients with RRAL undergoing allo-HSCT. Our results revealed that delaying pDLI time to day +90 based on MRD could lower extensive cGVHD incidence and improve GRFS without increasing incidence of leukemia relapse.

Currently, timing of pDLI is typically based on post-transplant MRD status (5, 6, 22, 23). For patients at high risk of relapse, some centers including ours have conducted pDLI without considering MRD status (7, 21, 24). Schmid et al. adopted the strategy of intensive chemotherapy, reduced-intensity conditioning and pDLI from day +120 in 12 patients with high-risk AML and myelodysplastic syndrome, with incidences of relapse and GVHD of 16.7% and 33.3% (21). Huang et al. demonstrated that pDLI was given at the median of 70 (range, 20–314) days post-transplant in 33 patients with advanced leukemia, with incidences of relapse and cGVHD of 45.5% and 62.5%, respectively (24). However, optimal timing of pDLI is uncertain. We previously adopted the strategy of pDLI on day +60 regardless of MRD test and then based on MRD and GVHD status from day +90 post-transplant in patients with RRAL, which was demonstrated to reduce relapse rate and improve survival (7). Nevertheless, the high incidence of extensive cGVHD after pDLI hindered survival and quality of life of patient (7). Consequently, in order to reduce the morbidity and mortality of GVHD, we modified our strategy of pDLI by postponing the infusion time to day +90 unless MRD was positive on day +60 and compared with previous pDLI strategy. Our results revealed that pDLI on day +90 post-transplant had a lower incidence of extensive cGVHD (10.3% vs. 27.9%) and superior GRFS (55.1% vs. 41.0%) than pDLI on day +60.

Except for the time interval from transplant to pDLI, other factors might also influence the morbidity and mortality of GVHD after pDLI such as the doses, HLA compatibility and donor source of pDLI (11, 12, 25). In general, risk of GVHD is lower in patients receiving pDLI from MSD, and higher in those receiving pDLI from MUD or HID (26, 27). However, some domestic studies including ours have shown that there are no significant differences in the morbidity and mortality of GVHD between patients receiving G-CSF-mobilized pDLI from MSD and HID (5, 28). It might be due to that the use of G-CSF might modulate polarization of T cells from Th1 to Th2 phenotype and indirectly induce T cell hypo-responsiveness and down-regulation of co-stimulatory signal of CD28/B7 (29, 30). In this study, we also observed that the morbidity and mortality of GVHD did not differ in the patients receiving pDLI from MSD and HID, which was consistent with our former finding (7).

Relapse is the major cause of death in patients with RRAL following transplant. Recently, some studies showed that the strategy of sequential intensified conditioning followed by pDLI could reduce leukemia relapse in patients with RRAL undergoing allo-HSCT (7, 21, 31). Schmid et al. reported that a sequential regimen of Flu/Ara-c/amsacrine chemotherapy and reduced-intensity conditioning along with immunosupressant withdrawal and pDLI were used for refractory AML undergoing allo-HSCT, with 2-year OS and leukemia mortality of 40.0% and 39.3% (21). In this study, we adopted the strategy of Flu/Ara-C salvage chemotherapy and TBI/CY/VP-16 myeloablative conditioning followed by early rapid tapering of immunosuppressant and modified pDLI, with 2-year OS and relapse rate of 64.1% and 28.2%. The favorable efficacy might be attributed to two aspects: salvage chemotherapy and myeloablative conditioning regimen decreased leukemia burden at the time of transplantation; early tapering of immunosuppressant combined with pDLI accelerated the GVL effect. In addition to disease status pre-transplant, genetics was another major cause of relapse post-transplant (32–34). Interestingly, in this study, unfavorable genetics was not a risk factor for relapse, which might be due to the fact that only patients with RRAL were enrolled in this study and most of them were accompanied by unfavorable genetics. Moreover, we also found that HID transplant was the protective factor for relapse, which was coherent with other studies (25, 35, 36).

There were some limitations in this study. Although this study was based on two prospective cohorts, they were non-parallel, which could not exclude the influence of factors such as improvement in medical technology and supportive treatment. Besides, no randomized studies have shown that pDLI is superior to non-pDLI. Therefore, large-scale and randomized controlled trials are needed to validate outcomes of patients undergoing non-pDLI and different pDLI strategies.

In conclusion, our study demonstrated that delaying pDLI to day +90 based on MRD can lower extensive cGVHD incidence and improve GRFS without increasing incidence of leukemia relapse for patients with RRAL undergoing allo-HSCT. This finding provides evidence for exploring optimal timing of pDLI in patients with RRAL undergoing allo-HSCT.

## Data Availability Statement

The raw data supporting the conclusions of this article will be made available by the authors, without undue reservation.

## Ethics Statement

The studies involving human participants were reviewed and approved by Nanfang Hospital-Ethics Committee. Written informed consent to participate in this study was provided by the participants’ legal guardian/next of kin.

## Author Contributions

QS, QL, and LX wrote the report and did the analysis. QL, LX, DN, DL, ZG, and ZJ designed the protocol. All authors contributed patients, provided clinical data, and revised and corrected the report. QL, DN, DL, ZG, and ZJ approved and recommended the protocol within each institute. All authors contributed to the article and approved the submitted version.

## Funding

This work was supported by the Natural Science Foundation of Guangdong Province (2019A1515011924), Project of the Zhujiang Science & Technology Star of Guangzhou City (No. 201806010029), and National Natural Science Foundation of China (No. U1401221, No. 81300445, No. 81470349, and No. 81770190).

## Conflict of Interest

The authors declare that the research was conducted in the absence of any commercial or financial relationships that could be construed as a potential conflict of interest.
